# Limitations of WTC Five-Year Assessment

**DOI:** 10.1289/ehp.115-a71

**Published:** 2007-02

**Authors:** Albert Miller

**Affiliations:** Saint Vincent Catholic Medical, Center-Queens, Jamaica, New York, E-mail: almiller@bqhcny.org

We have learned much about the respiratory disorders since the exposures of responders at the World Trade Center (WTC) site, especially from the publications of Prezant and colleagues about the presentations, follow-up, and impairments of pulmonary function and bronchial reactivity of the fire fighters and emergency medical technicians of the New York City Fire Department (Banauch et al. 2003, 2005, 2006; Prezant et al. 2002). These reports are especially informative because of the availability of preexposure clinical and spirometric data.

We appreciate the report of much-awaited results among 9,442 workers from the WTC Worker and Volunteer Medical Screening Program (Herbert et al. 2006). Because of the potential for major illness, the large number of subjects at risk, and the resultant enormous public interest, it is important that the information reported be properly understood. A number of limitations in this report must be pointed out.

Although the title identified this report (Herbert et al. 2006) as a 5-year assessment, screening examinations were performed between 16 July 2002 and 16 April 2004, < 1 year through < 3 years after 11 September 2001. There were no follow-up examinations, either at the 5-year or at any other interval.

Summary conclusions (Herbert et al. 2006), heavily reported in the media, lump all respiratory symptoms:

… 69% reported new or worsened respiratory symptoms while performing WTC work. Symptoms persisted to the time of examination in 59% of these workers.

The 69% with “any respiratory symptom” included 23.3% with no “lower respiratory symptoms.” A far smaller percentage of all workers (17.3%) complained of what may be considered the most important respiratory symptom, dyspnea, which was not quantified by any standard scale. Such a reliance on symptoms is subject to recall biases both for symptoms present before 9/11 and for the onset, worsening, and persistence of symptoms after 9/11.

Because physical examination and chest radiographs were unrevealing (Herbert et al. 2006), the only objective results were from pulmonary function tests. These were confined to spirometry, which does not provide insight into all aspects of respiratory impairment. The data presented by Herbert et al. (2006) are limited. Mean values for subsets (classified by WTC exposure, previous smoking history, etc.) are not given. Despite the frequency of cough (42.8%), wheeze (15.1%), and chest tightness (15.4%) and the common diagnoses of asthma/reactive airways dysfunction, only 7.6% of all responders showed airway obstruction, defined as a ratio of forced expiratory volume in 1 sec (FEV_1_) to forced vital capacity (FVC) less than the 5th percentile of the reference population. Unlike virtually all spirometric surveys of a large population (reviewed by Miller et al. 1991), Herbert et al. (2006) found little difference in impairment by smoking status. Most spirometric impairments were classified as restrictive, uncharacteristic of the symptoms and clinical diagnoses. This frequency of low FVC (22.7%) raises several issues: *a*) the effects of other clinical factors not reported on, such as obesity; *b*) technical considerations in subject performance or technician monitoring of the FVC maneuver, despite the investigators attention to these; and *c*) the appropriateness of the reference-predicted values.

We await further information and follow-up from these investigators, including results of additional diagnostic procedures not included in routine screening. These include a wider array of pulmonary function tests (full lung volumes, diffusing capacity), measurement of bronchial reactivity, computed tomography scans, and—in appropriate patients—bronchoalveolar lavage and lung biopsies, which would truly elucidate the respiratory disorders following WTC exposure.
